# Bone Marrow Soluble Immunological Mediators as Clinical Prognosis Biomarkers in B-Cell Acute Lymphoblastic Leukemia Patients Undergoing Induction Therapy

**DOI:** 10.3389/fonc.2021.696032

**Published:** 2021-09-27

**Authors:** Marlon Wendell Athaydes Kerr, Fábio Magalhães-Gama, Hiochelson Najibe Santos Ibiapina, Fabíola Silva Alves Hanna, Lilyane Amorim Xabregas, Eliana Brasil Alves, João Paulo Diniz Pimentel, Maria Perpétuo Socorro Sampaio Carvalho, Andréa Monteiro Tarragô, Andréa Teixeira-Carvalho, Olindo Assis Martins-Filho, Allyson Guimarães da Costa, Adriana Malheiro

**Affiliations:** ^1^ Programa de Pós-Graduação em Ciências Aplicadas à Hematologia, Universidade do Estado do Amazonas (UEA), Manaus, Brazil; ^2^ Diretoria de Ensino e Pesquisa, Fundação Hospitalar de Hematologia e Hemoterapia do Amazonas (HEMOAM), Manaus, Brazil; ^3^ Programa de Pós-Graduação em Imunologia Básica e Aplicada, Universidade Federal do Amazonas (UFAM), Manaus, Brazil; ^4^ Programa de Pós-Graduação em Ciências da Saúde, Instituto René Rachou - Fundação Oswaldo Cruz (FIOCRUZ) Minas, Belo Horizonte, Brazil; ^5^ Grupo Integrado de Pesquisas em Biomarcadores de Diagnóstico e Monitoração, Instituto René Rachou - FIOCRUZ Minas, Belo Horizonte, Brazil; ^6^ Programa de Pós-Graduação em Medicina Tropical, UEA, Manaus, Brazil; ^7^ Instituto de Pesquisa Clínica Carlos Borborema, Fundação de Medicina Tropical Dr. Heitor Vieira Dourado (FMT-HVD), Manaus, Brazil; ^8^ Escola de Enfermagem de Manaus, UFAM, Manaus, Brazil

**Keywords:** leukemia, bone marrow microenvironment, chemokines, cytokines, biomarkers

## Abstract

Different factors are used as predictors of unfavorable clinical outcomes in B-Cell Acute Lymphoblastic Leukemia (B-ALL) patients. However, new prognostic markers are needed in order to allow treatment to be more accurate, providing better results and an improved quality of life. In the present study, we have characterized the profile of bone marrow soluble mediators as possible biomarkers for risk group stratification and minimal residual disease (MRD) detection during induction therapy. The study featured 47 newly-diagnosed B-cell acute lymphoblastic leukemia (B-ALL) patients that were categorized into subgroups during induction therapy according to risk stratification at day 15 [Low Risk (LR), Low Risk increasing to High Risk (LR→HR) and High Risk (HR)] and the MRD detection on day 35 (MRD^(-)^ and MRD^(+)^). Soluble immunological mediators (CXCL8, CCL2, CXCL9, CCL5, CXCL10, IL-1β, IL-6, TNF, IFN-γ, IL-17A, IL-4, IL-5, IL-10 and IL-2) were quantified by cytometric bead array and ELISA. Our findings demonstrated that increased levels of CCL5, IFN-γ and IL-2 at baseline appeared as putative candidates of good prognosis in LR and MRD^(-)^ subgroups, while CCL2 was identified as a consistent late biomarker associated with poor prognosis, which was observed on D35 in HR and MRD^(+)^ subgroups. Furthermore, apparently controversial data regarding IL-17A and TNF did not allow the definition of these molecules as either positive or negative biomarkers. These results contribute to the search for novel prognostic indicators, and indicate the potential of bone marrow soluble mediators in prognosis and follow-up of B-ALL patients during induction therapy.

## Introduction

B-cell acute lymphoblastic leukemia (B-ALL) is the most common pediatric cancer in the world, and derives from the abnormal proliferation of immature malignant cells in the bone marrow (1). The outcome of pediatric B-ALL has improved significantly with current therapies, and these therapies have led to approximately 90% event-free survival of most patients (2–4). However, a considerable number of B-ALL patients still suffer relapses and the exposure to ineffective and potentially toxic therapies may lead to long-term side effects and morbidity.

The identification of predictive biomarkers of chemotherapy response aid in the establishment of alternative or more assertive early therapeutic interventions, and would reduce the duration and potential toxicity of long-term therapies. Ultimately, prognosis biomarkers provide better results and higher quality of life for B-ALL patients. In this context, detailed investigation of the bone marrow leukemic microenvironment, especially the network of soluble immunological mediators, offers an opportunity to discover novel potential biomarkers.

The soluble immunological mediators support the crosstalk between hematopoietic cells and bone marrow stromal cells, allowing for the dynamic nature of the hematopoietic compartment, responsible for the healthy production and replenishment of blood cells (5). However, the imbalance in the immunological mediators has been pointed out as hallmarks of acute leukemia (6–8). The presence of malignant cells outcompetes normal hematopoietic cells, leading to remodeling the hematopoietic microenvironment and the hijack of the network of mediators to support leukemogenesis, cell survival, proliferation and chemoresistance (9–13). Among the immunological mediators, several chemokines and cytokines have been shown to play a relevant role in the development and progression of cancer (14, 15).

The aim of the present investigation was to characterize the profile of soluble immunological mediators in bone marrow plasma from newly diagnosed B-ALL patients and identify potential biomarkers for risk group stratification and detection of minimal residual disease (MRD). The data presented here demonstrate that the high-risk subgroup and those with MRD detected upon induction therapy share a common chemokine/cytokine profile at baseline, indicating putative soluble mediators as prognostic biomarkers during induction chemotherapy. Furthermore, the analysis of alterations in bone marrow biomarkers during induction therapy revealed alternative biomarkers during late follow-up of B-ALL patients.

## Materials and Methods

### Ethical Statement

The present study was submitted to and approved by the Ethical Committee at Fundação Hospitalar de Hematologia e Hemoterapia do Amazonas (HEMOAM), under the protocol registration number #739.563/2014. The parents or legal guardians read and signed the informed consent form prior to inclusion of the patients in the study. The study fulfills the principles of the Helsinki declaration and the 466/2012 resolution of the Brazilian National Health Council for research involving human participants.

### Study Population

This study was carried out from August 2014 to March 2018 at Fundação HEMOAM, which is the state reference center for diagnosis and treatment of hematological diseases, and is located in Manaus, Amazonas state, Brazil.

**Table 1** summarizes the demographical and clinical features of the study population. The study enrolled a total do 47 patients with newly diagnosed common B-cell Acute Lymphoblastic Leukemia (B-ALL), of either gender (34 males and 13 females, median age=5 years old; interquartile range (IQR)=3-10). The diagnosis was performed according to the classification criteria and guidelines of the World Health Organization (16). The patients were categorized into three subgroups according to the risk group stratification for induction therapy on day 15 (D15), and these are denominated LR – Low Risk (n=19, 15 males and 4 females; median age=4 years old; IQR=3-6); LR→HR – Low Risk increasing to High Risk (n=5, 2 males and 3 females; median age=3 years old; IQR=2-4) and HR – High Risk (n=23, 17 males and 6 females; median age=10 years old; IQR=4-15). Additionally, the study population was also classified according to their minimal residual disease (MRD) detection by flow cytometry (reference value: <0,01%) on day 35 (D35), denominated as MRD^(-)^ – absence of MRD (n=14, 10 males and 4 females; median age=5 years old; IQR=3-9) and MRD^(+)^ – presence of MRD (n=33, 24 males and 9 females; median age=5 years old; IQR=3-10).

**Table 1:** Demographical and clinical features of the study population.

Groups	n	Gender	Age*
**ALL**	47	34M/13F	5 (3-10)
**Treatment Risk Stratification**			
**Low Risk (LR)**	19	15M/4F	4 (3-6)
**Low Risk→High Risk on D15 (LR→HR)**	5	2M/3F	3 (2-4)
**High Risk (HR)**	23	17M/6F	10 (4-15)^#^
**Minimal Residual Disease (MRD) on D35**			
**MRD^(-)^**	14	10M/4F	5 (3-9)
**MRD^(+)^**	33	24M/9F	5 (3-10)

*Age is reported in median (IQR). ^#^significant differences at p < 0.05 when compared to LR and LR→HR.

### Clinical Data Collection and Bone Marrow Sampling

Clinical data were collected from medical records kept at the Medical & Statistical Service, as well as from the Hematology Laboratory records. Bone marrow samples were obtained by iliac crest aspiration at three consecutive time points during the induction therapy in accordance with the protocols and guidelines of the Brazilian Group for Treatment of Childhood Leukemia – GBTLI-LLA-2009 (17), and denominated as D0 – at diagnosis baseline; D15 – during the induction therapy; and D35 – at the end of the induction therapy. Immediately after aspiration, the bone marrow samples were transferred to EDTA vacuum tubes (BD Vacutainer^®^ EDTA K2) and submitted to centrifugation at 900 *x* g, for 15 min at 4°C, to recover approximately 1 mL of bone marrow plasma in the supernatant. Samples were aliquoted and stored at -80°C until processing for analysis of soluble immunological mediators.

### Quantification of Soluble Immunological Mediators in Bone Marrow Plasma

A range of soluble immunological mediators, including chemokines, cytokines and growth factors (CXCL8, CCL2, CXCL9, CCL5, CXCL10, IL-6, TNF, IFN-γ, IL-17A, IL-4, IL-10 and IL-2) were quantified in the bone marrow microenvironment using cytometric bead array (CBA) (BD™ Human Chemokine and BD™ Human Cytokine Th1/Th2/Th17 kits, San Diego, CA, USA) assays, according to the manufacturer’s instructions. Samples were run in a FACS CantoII (BD^®^ Biosciences, San Jose, CA, USA) and the FCAP-Array software v3 (Soft Flow Inc., USA) was used for data analysis. Data were reported as mean fluorescence intensity (MFI) as well as concentration, according to the standard curves provided in the kits. The quantification of IL-1β and IL-5 was carried out by ELISA (BD™ Human OptEIA™ Set II, San Diego, CA, USA), and data were reported in picograms per milliliter (pg/mL) concentration according to the manufacturer’s instructions.

### Statistical and Biomarker Signature Analysis

Comparative analysis between subgroups was carried out using the Student’s t test or Mann-Whitney test. Multiple comparisons among subgroups were performed using one-way ANOVA followed by the Tukey or Kruskal-Wallis tests followed by Dunn’s test. In all cases, significance was considered at p<0.05. The GraphPad Prism Software v8.0.1 (San Diego, CA, USA) was used for statistical analysis. GraphPad Prism, Microsoft Excel and PowerPoint software were used for graphical arts.

Biomarker signature analysis was carried out according to Ibiapina et al. (18), by converting the original results of each variable expressed as a continuous variable into categorical data. The cut-off point to converter data were chosen based on the global median values of each soluble mediator, considering the data set that included all samples. These values were employed to classify the patients for each subgroup as they present “High” (above the cut-off) or “Low” (below the cut-off) of a given soluble mediator. The following cut-offs were used: (CXCL8 = 1039; CCL2 = 2062; CXCL9 = 9538; CCL5 = 48086; CXCL10 = 12655; IL-6 = 264; TNF=103; IFN-γ=98; IL-17A=100; IL-4 = 179; IL-10 = 188; IL-2 = 167 expressed in MFI and IL-1β=134; IL-5 = 289 expressed in pg/mL). The biomarker signatures were assembled in radar charts using the 50th percentile as a threshold to identify biomarkers with increased levels in a higher proportion of patients, and these were further selected for Venn diagram analysis (http://bioinformatics.psb.ugent.be/webtools/Venn/).

## Results

### Bone Marrow Soluble Mediators in B-ALL Patients According to the Risk Group Stratification on D15 of Induction Therapy

**Figure 1:**
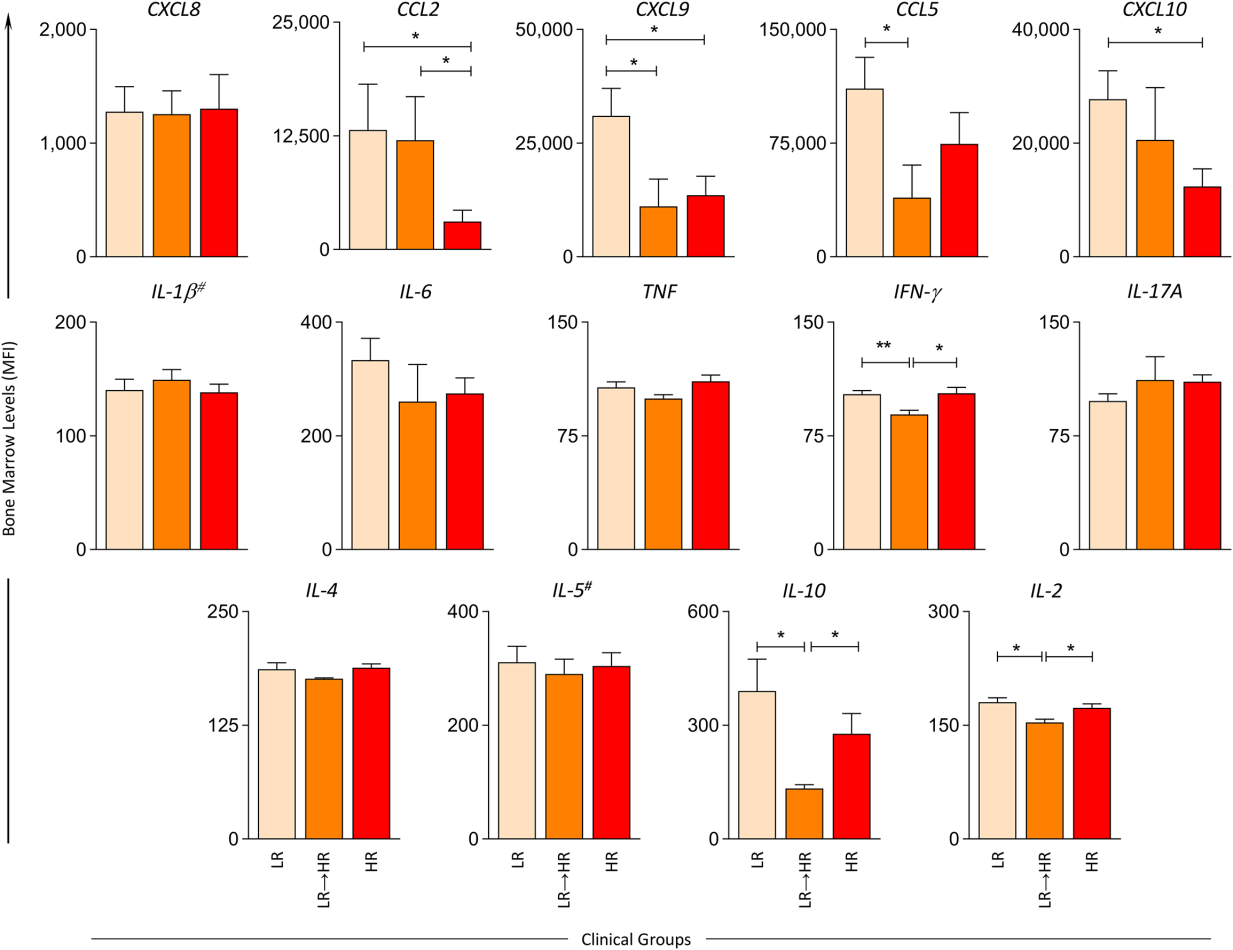
Bone marrow soluble mediators in B-ALL patients according to the risk group stratification on D15. The levels of bone marrow soluble mediators (CXCL8, CCL2, CXCL9, CCL5, CXCL10, IL-1β^#^, IL-6, TNF, IFN-γ, IL-17A, IL-4, IL-5^#^ IL-10, IL-2) were measured at the baseline (D0) in B-ALL patients categorized according to their risk group stratification on D15 of induction therapy, denominated as LR (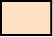), LR→HR (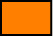) and HR (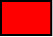) subgroups. Soluble mediators were quantified by cytometric bead array (CBA) and ELISA (IL-1β and IL-5)^#^ assays as described in the Materials and Methods section. Data are reported as mean concentration ± standard error expressed in mean fluorescence intensity (MFI) or pg/mL (IL-1β and IL-5). Statistical analyses were performed using Student’s t test or the Mann Whitney test and significant differences are underscored by connecting lines and asterisks for p < 0.01 (**) or p < 0.05 (*).

Aiming at characterization of the immunological profile in the bone marrow microenvironment and its impact on the prognosis of B-ALL response to induction therapy, patients were categorized according to risk group stratification (LR, LR→HR and HR) and the chemokine/cytokine levels were measured at diagnosis baseline. The results are reported in **Figure 1**. The results demonstrated that in general, the LR subgroup presented higher levels of bone marrow soluble mediators when compared to LR→HR (CXCL9, CCL5, IFN-γ, IL-10 and IL-2) and HR (CCL2, CXCL9, CXCL10). Conversely, the LR→HR displayed lower levels of CXCL9, CCL5 when compared to LR, as well as lower levels of IFN-γ, IL-10 and IL-2 when compared to both LR and HR (**Figure 1**). Detailed description of the results is provided in **Supplementary Table 1**.

### Overall Signature of Bone Marrow Soluble Mediators in B-ALL Patients According to the Risk Group Stratification on D15 of Induction Therapy

**Figure 2:**
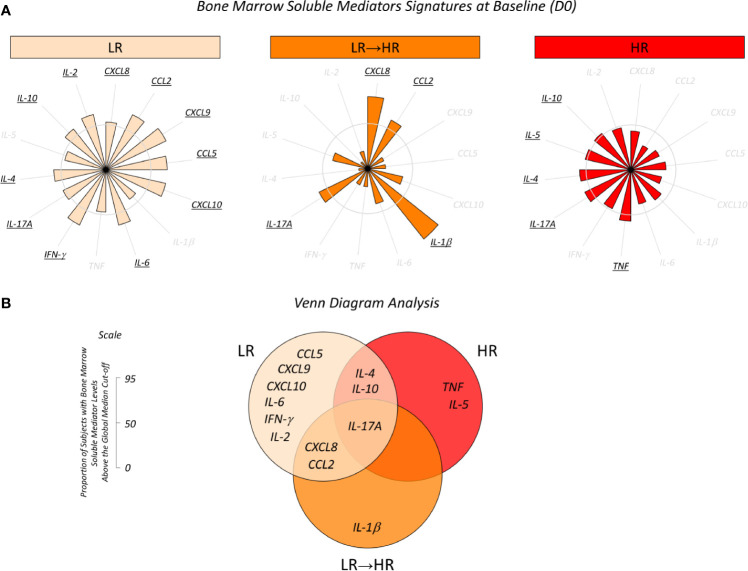
Overall signature of bone marrow soluble mediators in B-ALL patients according to the risk group stratification on D15. **(A)** Bone marrow soluble mediator signature at baseline (D0) in B-ALL patients categorized according to their risk group stratification on D15 of induction therapy, denominated as LR (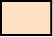), LR→HR (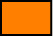) and HR (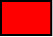) subgroups. Bone marrow soluble mediators were quantified using CBA and ELISA (IL-1β and IL-5) assays as described in the Materials and Methods section. Data, originally expressed in MFI or pg/mL (IL-1β and IL-5) were converted into categorical data using the global median values obtained for each biomarker as the cut-off to classify each patient since they presented low or high levels of immunological mediators in the bone marrow plasma. The biomarker signatures were assembled in radar charts using the 50^th^ percentile as the threshold (central circle) to identify biomarkers with increased levels in a higher proportion of patients, and then selected for further analysis. **(B)** Venn diagram analysis underscores common and selective biomarkers for each clinical subgroup with the potential to classify risk group stratification during induction.

Additional analysis was carried out to identify putative prognostic biomarkers for risk group stratification at D15 of induction therapy. For this purpose, the original results, which were expressed as a continuous variable, were converted into categorical data in order to estimate the proportion of patients with high levels of bone marrow soluble mediators and their relationship to risk group stratification, as presented in the **Figure 2**. Data mining was first performed by selecting the set of biomarkers observed in more than 50% of patients in each clinical subgroup. We observed that the LR subgroup exhibited a more robust profile of biomarkers, and these comprised high levels of CXCL8, CCL2, CXCL9, CCL5, CXCL10, IL-6, IFN-γ, IL-17A, IL-4, IL-10 and IL-2. Conversely, the LR→HR subgroup presented higher levels of CXCL8, CCL2, IL-1β and IL-17A. On the hand, the HR subgroup displayed lower levels of all chemokines evaluated, along with a minor increase in TNF, IL-17A, IL-4, IL-5 and IL-10 (**Figure 2A**).

Once the sets of biomarkers for LR, LR→HR and HR had been selected, Venn diagram analysis was carried out to identify the common and selective attributes amongst each clinical subgroup (**Figure 2B**). The analysis of intersections evidenced that IL-17A was a common biomarker observed in all patients, regardless of the risk group stratification. While IL-1β was identified as a selective attribute for LR→HR, and TNF and IL-5 for HR, a broader range of biomarkers was identified selectively in LR, and included CCL5, CXCL9, CXCL10, IL-6, IFN-γ and IL-2 (**Figure 2B**).

### Kinetics of Baseline Fold Changes on Bone Marrow Soluble Mediators From B-ALL Patients According to Induction Therapy Risk Group Stratification

**Figure 3:**
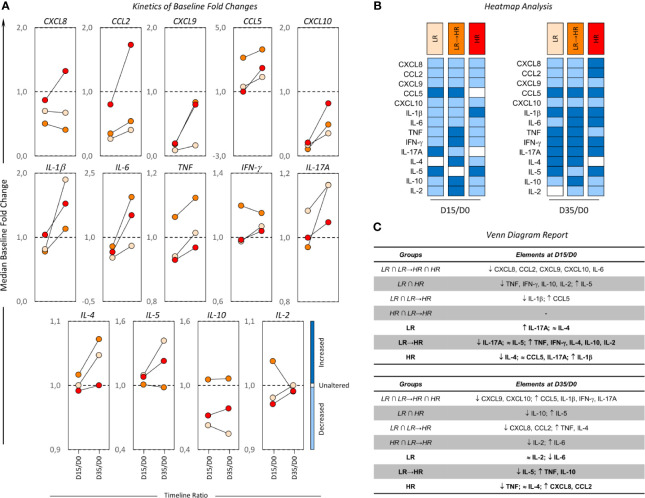
Kinetics of baseline fold changes on bone marrow soluble mediators from B-ALL patients according to their risk group stratification. **(A)** The median ratio of biomarker levels detected on D15 and D35 in relation to the baseline was determined for B-ALL patients categorized according to their risk group stratification on D15 of induction therapy are as follows: LR (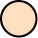), LR→HR (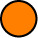) and HR (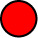) subgroups. Changes in biomarker levels on D15 and D35 were analyzed since they exhibited increased (baseline fold >1), unaltered (baseline folds=1) or decreased (baseline fold <1) levels in relation to D0. **(B)** The heat map compiles the changes in bone marrow biomarkers to identify the behavior patterns of soluble mediators in clinical subgroups. **(C)** Venn diagrams identify common and selective biomarkers for each clinical subgroup.

In order to determine the overall kinetics of bone marrow soluble mediators during induction therapy, the B-ALL patients were categorized according to induction therapy risk group stratification and the baseline fold changes evaluated on D15 and D35. The results are presented in **Figure 3** as the median ratio of biomarker levels detected on D15 and D35 in relation the baseline. Changes in biomarker levels on D15 and D35 were analyzed since they exhibited increased (baseline fold >1), unaltered (baseline folds=1) or decreased (baseline fold <1) levels in relation to D0 (**Figure 3A**). Heat map diagrams (**Figure 3B**) were used to compile the alterations in bone marrow biomarkers, and Venn diagrams (**Figure 3C**) were employed to identify common and selective alterations in each clinical subgroup. On D15, the data demonstrated an overall decrease in biomarkers (CXCL8, CCL2, CXCL9, CXCL10, IL-6) to be a common event, regardless of the risk group stratification. Selective increases in IL-17A were observed in LR, while a decrease in IL-5 and an increase in IL-1β were the hallmark of HR. It was notable that LR→HR exhibited decreased levels of IL-17A and increased levels of TNF, IFN-γ, IL-4, IL-10, IL-2. On D35, a general upregulation of biomarkers (CCL5, IL-1β, IFN-γ, IL-17A) was observed despite the risk group stratification. Selective changes were observed in LR, namely decreased levels of IL-6, and HR presented decreased levels of TNF and increased levels of CXCL8, CCL2. The LR→HR exhibited decreased levels of IL-5 and increased levels of TNF and IL-10 (**Figure 3C**).

### Bone Marrow Soluble Mediators in B-ALL Patients Classified According to the Minimal Residual Disease Detection on D35

**Figure 4:**
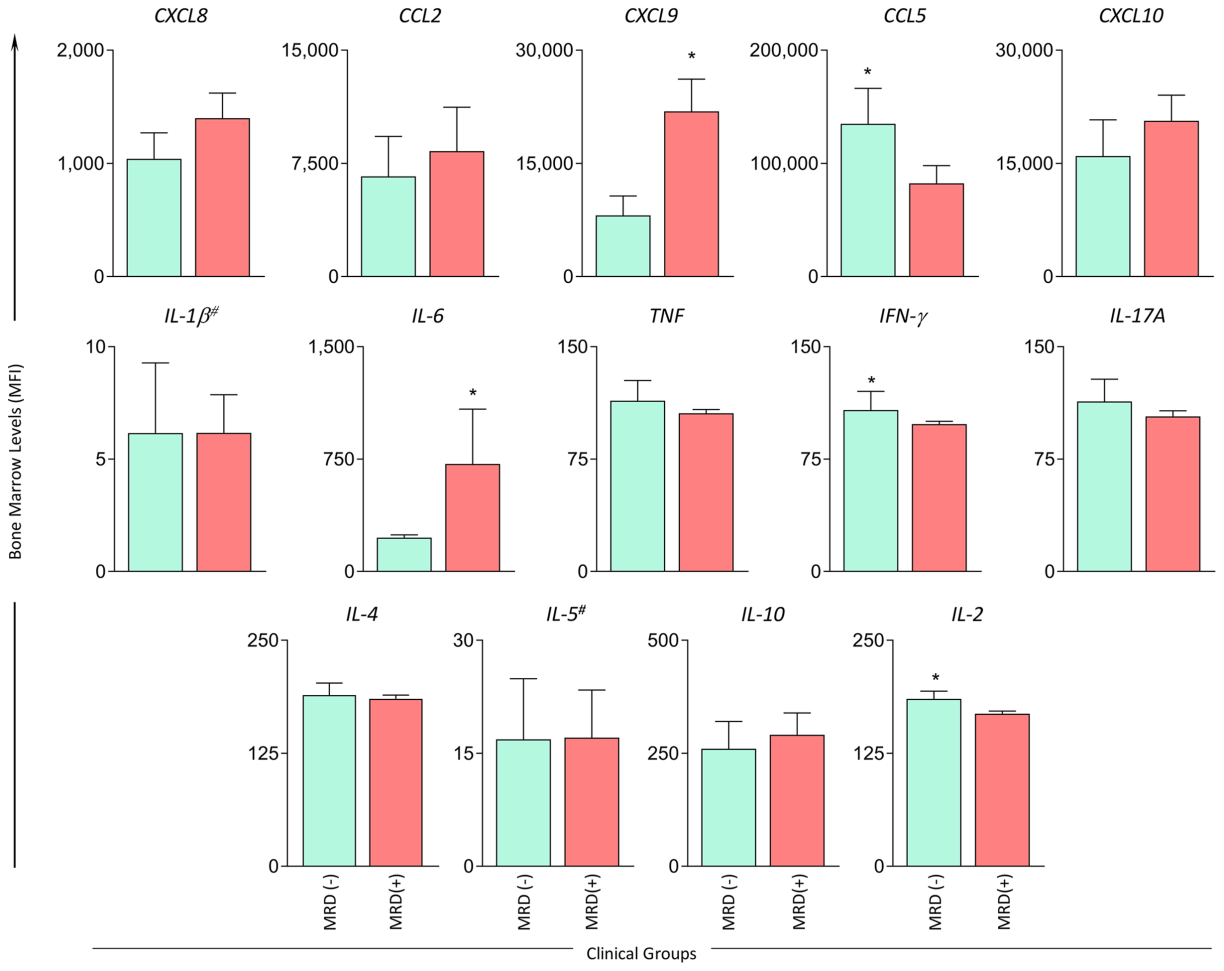
Bone marrow soluble mediators in B-ALL patients according to the minimal residual disease detection on D35. The levels of bone marrow soluble mediators (CXCL8, CCL2, CXCL9, CCL5, CXCL10, IL-1β^#^, IL-6, TNF, IFN-γ, IL-17A, IL-4, IL-5^#^ IL-10, IL-2) were measured at baseline (D0) in B-ALL patients categorized according to minimal residual disease detection on D35 of induction therapy, denominated as MRD^(-)^ (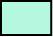) and MRD^(+)^ (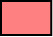) subgroups. Soluble mediators were quantified using CBA and ELISA (IL-1β and IL-5)^#^ assays as described in the Materials and Methods section. Data are reported as mean concentration ± standard error expressed in MFI or pg/mL (IL-1β and IL-5). Statistical analyses were performed using the Student’s t test or Mann Whitney test and significant differences are underscored by asterisks for p<0.05 (*).

The bone marrow biomarker profile was characterized in B-ALL patients according to MRD detection on D35 of induction therapy (MRD^(-)^ and MRD^(+)^) and the data is presented in **Figure 4**. A detailed description of results is provided in **Supplementary Table 2**. The results demonstrated that the MRD^(-)^ subgroup presented higher levels of CCL5, IFN-γ and IL-2 when compared to MRD^(+)^. On the other hand, the MRD^(+)^ subgroup presented higher levels of CXCL9 and IL-6 when compared to MRD^(-)^ (**Figure 4**).

### Overall Signature of Bone Marrow Soluble Mediators in B-ALL Patients According to the Minimal Residual Disease Detection on D35

**Figure 5:**
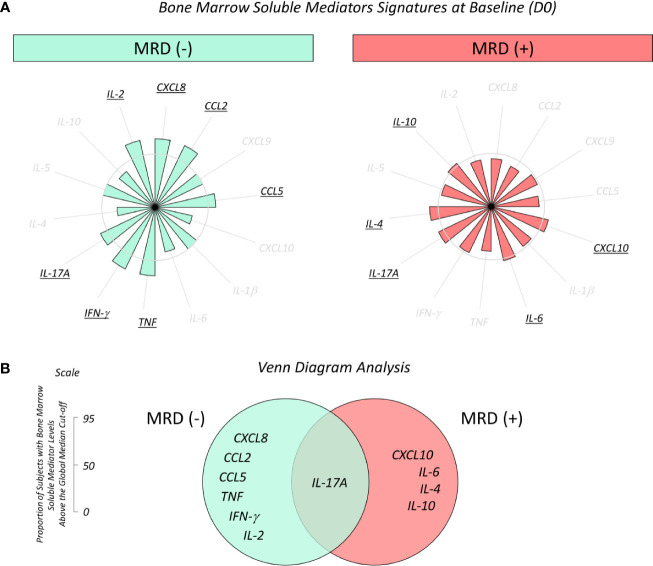
Overall signature of bone marrow soluble mediators in B-ALL patients according to the minimal residual disease detection on D35. **(A)** Bone marrow soluble mediator signature at baseline (D0) in B-ALL patients categorized according to the minimal residual disease detection on D35 of induction therapy, denominated as MRD^(-)^ (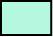) and MRD^(+)^ (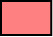) subgroups. Bone marrow soluble mediators were quantified by CBA and ELISA (IL-1β and IL-5) assays as described in the Materials and Methods section. Data, originally expressed in MFI or pg/mL (IL-1β and IL-5) were converted into categorical data using the global median values obtained for each biomarker as the cut-off to classify each patient since they presented low or high levels of immunological mediators in the bone marrow plasma. The biomarker signatures were assembled in radar charts using the 50^th^ percentile as the threshold (central circle) to identify biomarkers with increased levels in a higher proportion of patients, and then selected for further analysis. **(B)** Venn diagram analysis underscores common and selective biomarkers for each clinical subgroup with the potential to classify B-ALL patients according to the minimal residual disease detection on D35 of induction therapy.

Biomarker signature analysis of bone marrow soluble mediators from B-ALL patients classified as MRD^(-)^ and MRD^(+)^ confirming the MRD status on D35 are shown in **Figure 5**. Data analysis demonstrated that the MRD^(-)^ subgroup exhibited high levels of CXCL8, CCL2, CCL5, TNF, IFN-γ, IL-17A and IL-2, while MRD^(+)^ presented a minor increase in CXCL10, IL-6, IL-17A, IL-4 and IL-10 (**Figure 5A**). Venn diagram analysis evidenced that IL-17A was a common biomarker in both subgroups (MRD^(-)^ and MRD^(+)^). However, CXCL10, IL-6, IL-4 and IL-10 were selectively higher in MRD^(+)^, CXCL8, CCL2, CCL5, TNF, IFN-γ and IL-2 were selectively higher in MRD^(-)^ (**Figure 5B**).

### Kinetics of Baseline Fold Changes on Bone Marrow Soluble Mediators From B-ALL Patients According to Minimal Residual Disease Detection on D35

**Figure 6:**
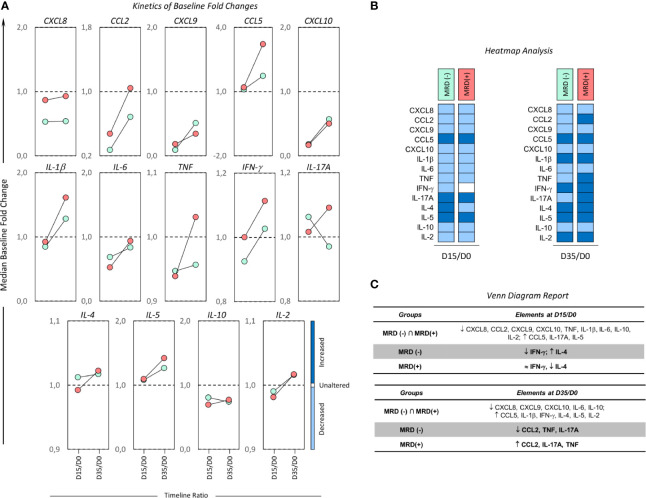
Kinetics of baseline fold changes on bone marrow soluble mediators from B-ALL patients according to minimal residual disease detection. **(A)** The median ratio of biomarker levels detected on D15 and D35 in relation to the baseline was determined for B-ALL patients categorized according to the minimal residual disease detection on D35 of induction therapy, as follows: MRD^(-)^ (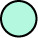) and MRD^(+)^ (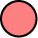) subgroups. Changes in biomarker levels on D15 and D35 were analyzed since they exhibited: increased (baseline fold >1), unaltered (baseline folds=1) or decreased (baseline fold <1) levels in relation to D0. **(B)** The heat map compiles the changes in bone marrow biomarkers to identify the behavior patterns of soluble mediators in clinical subgroups. **(C)** Venn diagrams identify common and selective biomarkers for each clinical subgroup.

The overall kinetics of the alterations in bone marrow soluble mediators in the MRD^(-)^ and MRD^(+)^ subgroups during induction therapy are presented in **Figure 6**. Changes in biomarker levels were analyzed considering the overall increased (baseline fold >1), unaltered (baseline folds=1) or decreased (baseline fold <1) levels in relation to D0 (**Figure 6A**). Heat map diagrams (**Figure 6B**) and Venn diagrams (**Figure 6C**) were employed to compile the overall profile and identify common and selective alterations in each clinical subgroup. The results demonstrated that on D15, a prominent decrease in biomarkers (CXCL8, CCL2, CXCL9, CXCL10, TNF, IL-1β, IL-6, IL-10, IL-2) was a common event, regardless of MRD detection. However, the data demonstrated on D15 that MRD^(-)^ subgroup presented a decrease in IFN-γ and an increase in IL-4, whereas MRD^(+)^ conversely exhibited a decrease in IL-4. On D35, a balance between upregulation (CXCL8, CXCL9, CXCL10, IL-6, IL-10) and downregulation (CCL5, IL-1β, IFN-γ, IL-4, IL-5, IL-2) of biomarkers was observed despite MRD detection. Of note was the finding that while the MRD^(-)^ subgroup presented a decrease in IL-2, TNF and IL-17A, MRD^(+)^ exhibited an increase in these molecules (**Figure 6C**).

## Discussion

The effectiveness of therapeutic intervention and survival of pediatric patients with acute lymphoblastic leukemia have improved in the last 5 decades mainly due to the concept of risk group stratification during induction therapy. Prognostic factors, such as age, leukocyte counts, cytogenetic findings and subtype of leukemia, have allowed us to make precise adjustments in the therapeutic schemes and have raised the survival rates from 10-20% to 80-90% (2–4, 19). In addition, the use of prognostic indicators, such as MRD detection during treatment, has contributed to improve therapeutic success in B-ALL patients. The detection of MRD can predict relapses after induction therapy since residual resistant leukemia cells are sources of recurrence and metastasis (20).

The signaling triggered by chemokines and cytokines is essential for homeostasis of the hematopoietic microenvironment, and disturbances in the chemokines and cytokines milieu may contribute to leukemogenesis and leukemia cell survival (9, 10, 21). Data regarding the complex network of soluble immunological mediators in the bone marrow of B-ALL patients still remains limited and represents a vast field for investigations. In this sense, the identification of predictive biomarkers for therapeutic response or MRD detection during induction therapy may contribute to the establishment of parameters for patient follow-up and more precise therapeutic intervention.

In the search for early biomarkers that can be identified at baseline and that have prognostic application for monitoring of B-ALL patients, our findings demonstrated that increased levels of CCL5, IFN-γ and IL-2 at baseline appeared as putative candidates of good prognosis in LR and MRD^(-)^ B-ALL patients (**Figures 1** and **4**). The analysis of biomarker signatures further supports these findings (**Figures 2** and **5**). It has been proposed that while cytokine levels may influence leukemia progression at multiple time points during disease development, other studies have suggested a relevant early influence of basal cytokine production on leukemogenesis (22). Nevertheless, studies of chemokines and cytokines in B-ALL that focus on prognostic markers are still scarce. Research using murine models have demonstrated that the absence of IFN-γ results in higher numbers of leukemia-initiating cells and accelerated leukemia onset (22).

IL-2 is a potent growth factor that plays a role supporting the T-cell response (23) and leads to the differentiation of CD4^+^ T-cells, the maintenance of regulatory T-cells and elicits the cytotoxic response of CD8^+^ T-cells and NK-cells (24, 25). In this sense, the increased levels of IL-2, together with IFN-γ observed in LR and MRD^(-)^, may support a potent and balanced T-cell response that, together with enhanced levels of CCL5, participate in the recruitment, activation and migration of CD4^+^ and CD8^+^ T-cells and NK-cells (26, 27), and ultimately favor the elimination of leukemia cells.

Importantly, the LR subgroup also presented high production of the anti-inflammatory cytokines IL-4 and IL-10, indicating that the balance between Th1 and Th2 cytokines profiles may be associated to a better prognosis in these patients. This reinforced by the lower levels of IFN-γ and IL-2 presented by the HR subgroup, which, unlike the LR, displayed a response triggered towards a Th2 profile with increased levels of IL-4, IL-5 and IL-10. Indeed, previous studies demonstrated that B-ALL pediatric patients show, at the time of diagnosis, an imbalance in the immunological profile with shading of pro-inflammatory cytokines associated with cytotoxic responses and increase in cytokines that, when overexpressed, inhibit antitumor responses (8, 28–31).

Our data demonstrated that B-ALL patients displayed higher levels of IL-17A at baseline regardless of the risk group stratification or MRD detection. Although Th17 cells are implicated in several oncologic diseases, their role in cancer has not been well elucidated and remains under debate (32–35). In this context, it is also important to consider that the Th17 cell subset and IL-17 are not synonymous. In cancer patients, besides IL-17, Th17 cells produce high levels of other immunological mediators, such as GM-CSF, IL-2, TNF and IFN-γ, but not IL-10. Perhaps the most conflicting data regarding the role of Th17 in cancer arises from studies examining IL-17 itself. IL-17 is a pro-inflammatory mediator that can induce the production of several pro-inflammatory cytokines and chemokines. Several studies have investigated the antitumor function of IL-17, but it is still difficult to infer the exact role that this cytokine has in cancer patients (33). Human studies are challenging, but they are essential in order to investigate the relationship between IL-17 at early phases of human oncologic conditions so that we may better understand their roles during disease progression.

During the kinetic evaluation of induction therapy, the B-ALL patients classified as Low Risk displayed a selective increase in IL-17A onD15. Conversely, our data also demonstrated that MRD^(+)^ presented increased levels of IL-17A on D35. Likewise, the TNF profile also demonstrated this contradictory pattern with increased levels at baseline observed in HR and MRD^(-)^ (**Figures 2** and **5**) and decreased levels were observed in both subgroups on D35 (**Figures 3** and **6**). These apparently controversial findings regarding the IL-17A and TNF profile during the kinetic evaluation did not allow the definition of these molecules as being either positive or negative biomarkers for prognosis of B-ALL.

The analysis of kinetics during induction therapy indicated that CCL2 is a consistent late biomarker associated with poor prognosis, and is observed on D35 in HR and MRD^(+)^ subgroups. Several studies have reported that CCL2 is highly expressed by cancer cells in solid neoplasms and oncohematological diseases (36–39). Studies of patients with acute myeloid leukemia have shown increased serum levels of CCL2 in comparison with healthy controls (38). Similar results have been observed in patients with chronic lymphocytic leukemia, which suggests that the CCL2 is associated with the persistence of leukemic cells (39, 40).

The present study has some limitations. Since the bone marrow microenvironment is formed by a robust and complex network of immunological mediators, it is important that a more complete assessment is performed, in which other chemokines and cytokines are quantified, together with other mediators and elements capable of playing a role as biomarkers. In addition, the reduced number of individuals included in the clinical subgroups evaluated may be a restriction for the interpretation of the results. However, it is important to consider the difficulty of working with pediatric populations and the barriers to conducting longitudinal investigations, especially as many pediatric patients have restrictions to follow the study design. We believe that additional studies with a large number of patients and a longer follow-up period are needed to demonstrate the precise role that certain molecules, such as IL-17A and TNF, play in B-ALL.

## Conclusion

**Figure 7:**
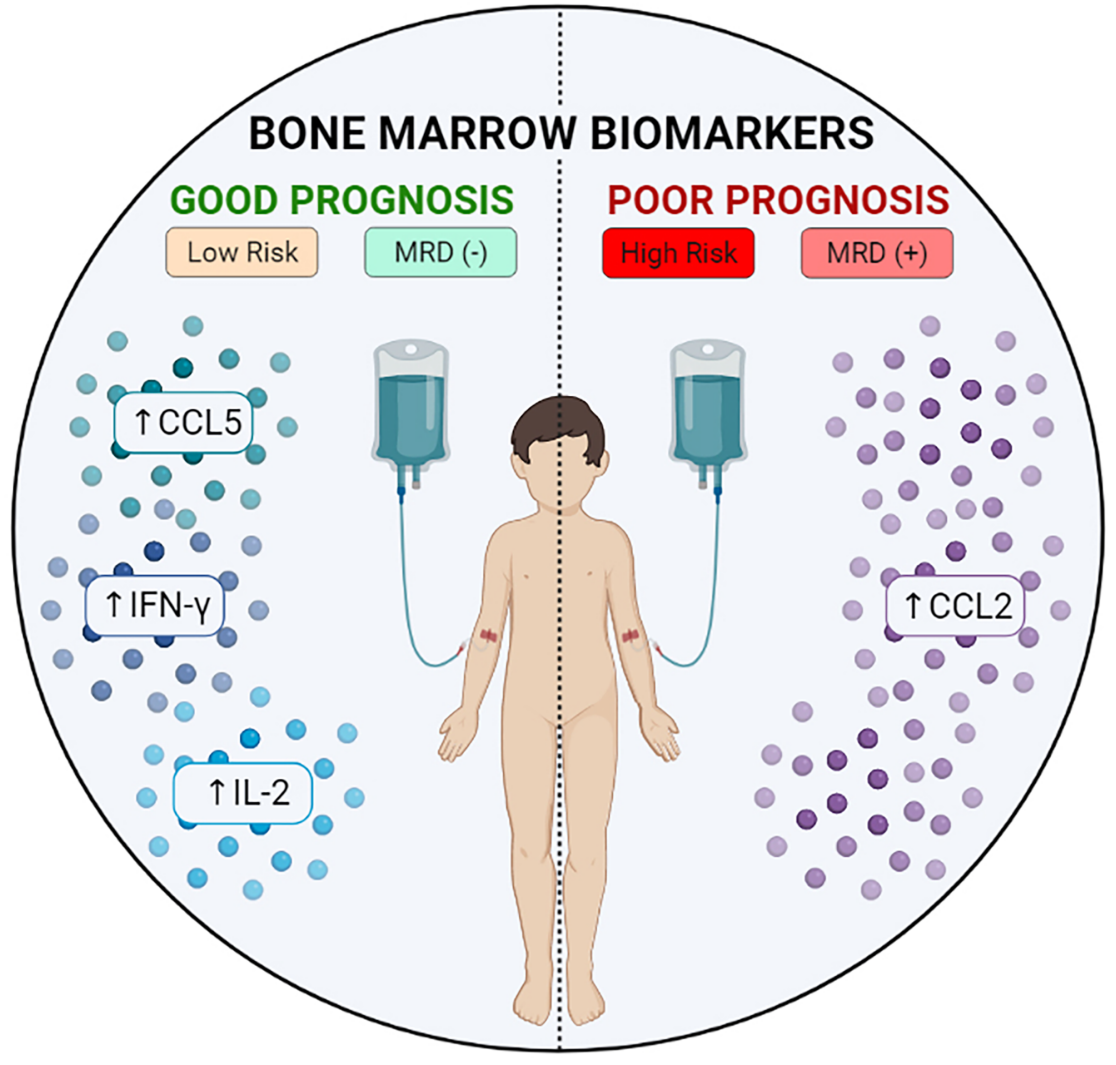
Potential bone marrow biomarkers during induction therapy. Schematic presentation summarizing the biomarkers of good or bad prognosis identified in the study.

In synthesis, our data demonstrated that increased levels of CCL5, IFN-γ and IL-2 at baseline appeared as putative candidates of good prognosis in LR and MRD^(-)^ subgroups, while CCL2 was identified as a consistent late biomarker associated with poor prognosis, which was observed on D35 in HR and MRD^(+)^ subgroups (**Figure 7**) and may indicate the need for alternative and more assertive therapeutic interventions in order to increase the effectiveness of induction therapy. Additionally, apparently controversial data regarding IL-17A and TNF during the kinetic evaluation did not allow the definition of these molecules as either positive or negative biomarkers. Our results contribute to the search for novel prognostic indicators, and suggest the potential of bone marrow soluble mediators in prognosis and follow-up of B-ALL patients during induction therapy.

## Data Availability Statement

The datasets presented in this study can be found in online repositories. The names of the repository/repositories and accession number(s) can be found in the article/**Supplementary Material**.

## Ethics Statement

The present study was submitted to and approved by the Ethical Committee at Fundação Hospitalar de Hematologia e Hemoterapia do Amazonas (HEMOAM), under the protocol registration number #739.563/2014. The parents or legal guardians read and signed the informed consent form prior to inclusion of the patients in the study. The study fulfills the principles of the Helsinki declaration and the 466/2012 resolution of the Brazilian National Health Council for research involving human participants. Written informed consent to participate in this study was provided by the participants’ legal guardian/next of kin.

## Author Contributions

MK, FM-G, OM-F, AC, and AM designed, performed the experiments, analyzed data, and wrote the manuscript. MK, FM-G, EA, JP, OM-F, and AC analyzed data. MK, FM-G, HI, FH, LX, EA, and AC recruited all individuals, performed the experiments, and revised the manuscript. MC, JP, AT, and AT-C revised the manuscript. MK, FM-G, OM-F, AC, and AM supervised the project development, designed the experiments, interpreted the data, wrote, and revised the manuscript. All authors contributed to the article and approved the submitted version.

## Funding

Financial support was provided in the form of grants from Fundação de Amparo à Pesquisa do Estado do Amazonas (FAPEAM) (Pró-Estado Program - #002/2008, #007/2018 and #005/2019, PAMEQ Program - #004/2019, PAPAC Program - #005/2019 and PECTI-AM/SAÚDE Program #004/2020), Conselho Nacional de Desenvolvimento Científico e Tecnológico (CNPq) and Coordenação de Aperfeiçoamento de Pessoal de Nível Superior (CAPES) (PROCAD-Amazônia 2018 Program - #88881.200581/2018-01). MK, FM-G, FH, and LX have fellowships from FAPEAM and CAPES (Master’s student). AT-C is a level 2 research fellow from CNPq and a research fellow from FAPEAM (PVN-II, PECTI-AM/SAÚDE Program #004/2020). OM-F is a level 1 research fellow from CNPq and a research fellow from FAPEAM (PVN-II, Pró-Estado Program - #002/2008, #007/2018 and #005/2019). AM is a level 2 research fellow from CNPq. The funders had no contribution in the study design, data collection and analysis, decision to publish or manuscript preparation.

## Conflict of Interest

The authors declare that the research was conducted in the absence of any commercial or financial relationships that could be construed as a potential conflict of interest.

## Publisher’s Note

All claims expressed in this article are solely those of the authors and do not necessarily represent those of their affiliated organizations, or those of the publisher, the editors and the reviewers. Any product that may be evaluated in this article, or claim that may be made by its manufacturer, is not guaranteed or endorsed by the publisher.

## Abbreviations

B-ALL, Acute Lymphoblastic Leukemia; CBA, cytometric bead array; D0, diagnosis baseline; D15, day 15 of the induction therapy; D35, the end of the induction therapy; HR, High Risk; IQR, interquartile range; LR, Low Risk; LR→HR, Low Risk increasing to High Risk; MFI, mean fluorescence intensity; MRD, minimal residual disease; MRD^(-)^, absence of MRD; MRD^(+)^, presence of MRD; NK, natural killer; Th, T helper.
