# Supplementary material for: Bone Marrow Soluble Immunological Mediators as Clinical Prognosis Biomarkers in B-Cell Acute Lymphoblastic Leukemia Patients Undergoing Induction Therapy
Source: Front Oncol. 2021 Sep 27;11:696032. doi: 10.3389/fonc.2021.696032 (PMC8503185; doi:10.3389/fonc.2021.696032)
Supplement: Supplementary file 1 [file Table_1.docx]

Supplementary Table 1. Kinetics of bone marrow soluble mediators in B-cell acute lymphoblastic leukemia patients according to the risk group stratification for induction therapy at D15.

| **Parameters^§^** |  | **Low Risk (LR)** | | | | |  | **Low Risk→High Risk (LR→HR)** | | | | |  | **High Risk (LR)** | | | | |
| --- | --- | --- | --- | --- | --- | --- | --- | --- | --- | --- | --- | --- | --- | --- | --- | --- | --- | --- |
|  |  | **D0** |  | **D15** |  | **D35** |  | **D0** |  | **D15** |  | **D35** |  | **D0** |  | **D15** |  | **D35** |
|  |  |  |  |  |  |  |  |  |  |  |  |  |  |  |  |  |  |  |
| **CXCL8** |  | 1,277  ±220 |  | 1,562  ±690 |  | 722  ±88 |  | 1,255  ± 204 |  | 798  ±204 |  | 572  ±91 |  | 2,546  ±1,276 |  | 764  ±112 |  | 1,322  ±274 |
| **CCL2** |  | 8,099  ±2,192 |  | 3,967  ±1,645 |  | 3,372  ±588 |  | 12,027  ±4,779 |  | 3,231  ±1,074 |  | ***19,837***  ***±13,333 **** |  | ***1,909***  ***±519 *^,#^*** |  | 1,094  ±197 |  | ***2,941***  ***±627 ^b,#^*** |
| **CXCL9** |  | 22,593  ±4,582 |  | ***2,679***  ***±672 ^a^*** |  | ***2,935***  ***±514 ^a^*** |  | 5,242  ±1,629 |  | ***1,088***  ***±203 ^a^*** |  | 2,756  ±890 |  | 13,551  ±4,166 |  | ***1,886***  ***±605 ^a^*** |  | ***4,466***  ***±694 ^a^*** |
| **CCL5** |  | 105,692  ±20,219 |  | 145,802  ±18,163 |  | 183,361  ±17,033^a^ |  | 39,085  ±21,458 |  | 84,040  ±17,846 |  | 129,865  ±38,434 |  | 82,688  ±21,236 |  | 109,077  ±19,183 |  | 138,478  ±16,450 |
| **CXCL10** |  | 26,279  ±4,932 |  | ***8,863***  ***±4,482 ^a^*** |  | ***6,613***  ***±1,390 ^a^*** |  | 5,693  ±2,647 |  | 2,275  ±902 |  | 5,967  ±613 |  | 13,152  ±3,063 |  | ***3,216***  ***±942 ^a^*** |  | 9,446  ±2,148 |
|  |  |  |  |  |  |  |  |  |  |  |  |  |  |  |  |  |  |  |
| **IL-1β^‡^** |  | 140  ±10 |  | 131  ±13 |  | ***277***  ***±24*** ***^a,b^*** |  | 179  ±31 |  | 132  ±17 |  | 174  ±26 |  | 138  ±7 |  | 150  ±14 |  | ***212***  ***±18 ^a,b^*** |
| **IL-6** |  | 333  ±38 |  | 709  ±511 |  | 234  ±34 |  | 260  ±65 |  | 167  ±10 |  | 266  ±49 |  | 275  ±27 |  | 200  ±50 |  | 391  ±66 ^b^ |
| **TNF** |  | 107  ±4 |  | 103  ±4 |  | 113  ±4 |  | 100  ±2 |  | 125  ±22 |  | 118  ±12 |  | 111  ±4 |  | 130  ±28 |  | 122  ±14 |
| **IFN**-**γ** |  | 103  ±2 |  | 96  ±2 |  | ***109***  ***±5 ^b^*** |  | 89  ±3 |  | 102  ±7 |  | 107  ±10 |  | 103  ±4 |  | 108  ±7 |  | 123  ±12 |
| **IL-17A** |  | 100  ±5 |  | 121  ±10 |  | 126  ±8 |  | 112  ±15 |  | 103  ±9 |  | 112  ±6 |  | 111  ±4 |  | 109  ±5 |  | 120  ±11 |
|  |  |  |  |  |  |  |  |  |  |  |  |  |  |  |  |  |  |  |
| **IL-4** |  | 187  ±7 |  | 188  ±7 |  | 187  ±5 |  | 176  ±1 |  | 184  ±8 |  | 200  ±13 |  | 188  ±4 |  | 203  ±22 |  | 206  ±16 |
| **IL-5^‡^** |  | 311  ±28 |  | 350  ±27 |  | ***466***  ***±42 ^a,b^*** |  | 290  ±26 |  | 297  ±35 |  | 345  ±76 |  | 304  ±23 |  | 335  ±25 |  | ***416***  ***±36 ^a^*** |
| **IL-10** |  | 280  ±51 |  | ***139***  ***±7 ^a^*** |  | ***131***  ***±5 ^a^*** |  | 133  ±10 |  | 133  ±9 |  | 136  ±6 |  | 232  ±29 |  | 160  ±29 |  | 185  ±26 |
|  |  |  |  |  |  |  |  |  |  |  |  |  |  |  |  |  |  |  |
| **IL-2** |  | 179  ±6 |  | 181  ±10 |  | 178  ±5 |  | 158  ±5 |  | 170  ±12 |  | 168  ±11 |  | 174  ±5 |  | 173  ±7 |  | 187  ±15 |
|  |  |  |  |  |  |  |  |  |  |  |  |  |  |  |  |  |  |  |

^§^Data are expressed in mean fluorescence intensity (MFI), expect for IL1-β^‡^ and IL-5^‡^ that are reported in pg/mL Significant differences at p<0,05 for intragroup analysis are underscored by letters “a” and “b” as compared to D0 and D15, respectively, Significant differences at p<0,05 for intergroup analysis are underscored by * and # as compared to LR and LR→HR, respectively.
