# Supplementary material for: Bone Marrow Soluble Immunological Mediators as Clinical Prognosis Biomarkers in B-Cell Acute Lymphoblastic Leukemia Patients Undergoing Induction Therapy
Source: Front Oncol. 2021 Sep 27;11:696032. doi: 10.3389/fonc.2021.696032 (PMC8503185; doi:10.3389/fonc.2021.696032)
Supplement: Supplementary file 2 [file Table_2.docx]

Supplementary Table 2. Kinetics of bone marrow soluble mediators in B-cell acute lymphoblastic leukemia patients according to the minimal residual disease detection on D35.

| **Parameters^§^** |  | **Negative minimal residual disease (MRD^-^)** | | | | |  | **Positive minimal residual disease (MRD^+^)** | | | | |
| --- | --- | --- | --- | --- | --- | --- | --- | --- | --- | --- | --- | --- |
|  |  | **D0** |  | **D15** |  | **D35** |  | **D0** |  | **D15** |  | **D35** |
|  |  |  |  |  |  |  |  |  |  |  |  |  |
| **CXCL8** |  | 1,643  ±442 |  | ***629***  ***±101 ^a^*** |  | 824  ±106 |  | 1,143  ±156 |  | 890  ±111 |  | 1,107  ±214 |
| **CCL2** |  | 7,558  ±2,437 |  | ***1,968***  ***±962 ^a^*** |  | 3,077  ±759 |  | 7,922  ±2,944 |  | 2,641  ±875 |  | 5,691  ±2,165 |
| **CXCL9** |  | 17,122  ±6,651 |  | ***1,149***  ***±434 ^a^*** |  | ***2,291***  ***±420 ^a^*** |  | 18,969  ±3,686 |  | ***2,535***  ***±533*** ***^a^*** |  | ***4,779***  ***±721 ^a,^**** |
| **CCL5** |  | 99,243  ±27,700 |  | 124,311  ±23,882 |  | 174,750  ±24,170 |  | 82,303  ±15,487 |  | ***119,966***  ***±14,538*** |  | ***147,626***  ***±12,941^a^*** |
| **CXCL10** |  | 17,034  ±5,861 |  | ***4,037***  ***±1,655 ^a^*** |  | 5,508  ±1,693 |  | 20,185  ±3,111 |  | ***5,976***  ***±2,608 ^a^*** |  | ***10,767***  ***±2,295 ^a^*** |
|  |  |  |  |  |  |  |  |  |  |  |  |  |
| **IL-1β**^‡^ |  | 147  ±14 |  | 123  ±9 |  | ***210***  ***±23*** ***^a,b^*** |  | 142  ±7 |  | 147  ±12 |  | ***245***  ***±17*** ***^a,b^*** |
| **IL-6** |  | 276  ±29 |  | 158  ±15 |  | 276  ±67 |  | 331  ±37 |  | 212  ±40 |  | ***450***  ***±92 ^b^*** |
| **TNF** |  | 116  ±6 |  | 115  ±9 |  | 109  ±4 |  | 105  ±2 |  | ***100***  ***±2 **** |  | 121  ±10 |
| **IFN-γ** |  | 106  ±4 |  | 99  ±2 |  | 105  ±4 |  | 100  ±3 |  | 104  ±5 |  | 112  ±4 |
| **IL-17A** |  | 116  ±8 |  | 115  ±5 |  | 123  ±8 |  | 102  ±3 |  | 112  ±7 |  | 121  ±8 |
|  |  |  |  |  |  |  |  |  |  |  |  |  |
| **IL-4** |  | 193  ±11 |  | 191  ±9 |  | 188  ±5 |  | 184  ±2 |  | 196  ±15 |  | 201  ±12 |
| **IL-5**^‡^ |  | 323  ±35 |  | 348  ±40 |  | 446  ±63 |  | 298  ±17 |  | 332  ±17 |  | ***422***  ***±26 ^a,b^*** |
| **IL-10** |  | 266  ±81 |  | 131  ±8 |  | 144  ±15 |  | 318  ±51 |  | ***155***  ***±20 ^a^*** |  | ***165***  ***±19 ^a^*** |
|  |  |  |  |  |  |  |  |  |  |  |  |  |
| **IL-2** |  | 180  ±6 |  | 184  ±13 |  | 177  ±6 |  | 172  ±4 |  | 172  ±6 |  | 183  ±10 |
|  |  |  |  |  |  |  |  |  |  |  |  |  |

^§^Data are expressed in MFI, expect for IL1-β^‡^ and IL-5^‡^, which are reported in pg/mL Significant differences at p<0,05 for intragroup analysis are underscored by letters “a” and “b” when compared to D0 and D15, respectively, Significant differences at p<0,05 for intergroup analysis are underscored by * when compared to MRD.
